# Uterine Cancer Incidence and Mortality — United States, 1999–2016

**DOI:** 10.15585/mmwr.mm6748a1

**Published:** 2018-12-07

**Authors:** S. Jane Henley, Jacqueline W. Miller, Nicole F. Dowling, Vicki B. Benard, Lisa C. Richardson

**Affiliations:** 1 Division of Cancer Prevention and Control, National Center for Chronic Disease Prevention and Health Promotion, CDC.

## Abstract

Uterine cancer is one of the few cancers with increasing incidence and mortality in the United States, reflecting, in part, increases in the prevalence of overweight and obesity since the 1980s (*1*). It is the fourth most common cancer diagnosed and the seventh most common cause of cancer death among U.S. women (*1*). To assess recent trends in uterine cancer incidence and mortality by race and ethnicity, CDC analyzed incidence data from CDC’s National Program of Cancer Registries (NPCR) and the National Cancer Institute’s Surveillance, Epidemiology, and End Results (SEER) program and mortality data from the National Vital Statistics System (*2*). Most recent data available are through 2015 for incidence and through 2016 for mortality. Uterine cancer incidence rates increased 0.7% per year during 1999–2015, and death rates increased 1.1% per year during 1999–2016, with smaller increases observed among non-Hispanic white (white) women than among women in other racial/ethnic groups. In 2015, a total of 53,911 new uterine cancer cases, corresponding to 27 cases per 100,000 women, were reported in the United States, and 10,733 uterine cancer deaths (five deaths per 100,000 women) were reported in 2016. Uterine cancer incidence was higher among non-Hispanic black (black) and white women (27 cases per 100,000) than among other racial/ethnic groups (19–23 per 100,000). Uterine cancer deaths among black women (nine per 100,000) were higher than those among other racial/ethnic groups (four to five per 100,000). Public health efforts to help women achieve and maintain a healthy weight and obtain sufficient physical activity can reduce the risk for developing cancer of the endometrium (the lining of the uterus), the most common uterine cancer. Abnormal vaginal bleeding, including bleeding between periods or after sex or any unexpected bleeding after menopause, is an important symptom of uterine cancer (*3*). Through programs such as CDC’s Inside Knowledge* campaign, promoting awareness among women and health care providers of the need for timely evaluation of abnormal vaginal bleeding can increase the chance that uterine cancer is detected early and treated appropriately.

Data on new cases of invasive uterine cancer^†^ diagnosed during 1999–2015 were obtained from population-based cancer registries affiliated with NPCR and SEER. Data from all registries met data quality criteria for U.S. Cancer Statistics^§^ in 2015, and data from 48 states met these criteria each year during 1999–2015, covering 98% of the U.S. population.^¶^ Uterine cancers were classified by histologic type (endometrioid carcinoma, other carcinoma, carcinosarcoma, and sarcoma).** Stage at diagnosis (localized, regional, distant, or unknown) was characterized using SEER Summary Stage.^††^ Data on uterine cancer deaths during 1999–2016 were based on death certificate information reported to state vital statistics offices and compiled into a national file through the National Vital Statistics System, covering 100% of the U.S. population. Data were examined by five mutually exclusive racial/ethnic groups: white, black, non-Hispanic American Indian/Alaska Native (AI/AN), non-Hispanic Asian/Pacific Islander (API), and Hispanic; as well as by histologic type, stage at diagnosis, and year of diagnosis or death.

Population estimates for rate denominators were a modification of annual county population estimates by age, sex, bridged-race, and ethnicity produced by the U.S. Census Bureau in collaboration with CDC’s National Center for Health Statistics and with support from the National Cancer Institute.^§§^ Annual incidence and death rates per 100,000 women were age-adjusted to the 2000 U.S. standard population. Average annual percent change (AAPC) was used to quantify changes in incidence rates during 1999–2015 and death rates during 1999–2016 and was calculated using joinpoint regression, which allowed different slopes for three periods; years at which slope changed could vary.^¶¶^ To determine whether AAPC was significantly different from zero, a t-test was used for zero joinpoints, and a z-test was used for ≥1 joinpoint. Rates were considered to increase if AAPC >0 (p<0.05) and to decrease if AAPC <0 (p<0.05); otherwise rates were considered stable. All statistical tests were two-sided.

In 2015, 53,911 new microscopically confirmed uterine cancer cases, corresponding to 27 cases per 100,000 women, were reported in the United States (Table). Uterine cancer incidence was higher among white women and black women (27 cases per 100,000 in each group) compared with AI/AN and Hispanic women (23 each) and API women (19). Overall, endometrioid carcinomas were the most common uterine cancers (68%). However, endometrioid carcinomas accounted for 47% of uterine cancers among black women, who had a higher percentage of other carcinomas, carcinosarcomas, and sarcomas than did women in other racial/ethnic groups. Approximately two thirds of uterine cancers were diagnosed at a localized stage among white (69%), AI/AN (68%), API (67%), and Hispanic women (66%), compared with 55% among black women. A higher proportion of sarcomas were diagnosed at distant stage (36%) than were endometrioid carcinomas (3%), other carcinomas (18%), or carcinosarcomas (22%). The proportion of uterine cancers diagnosed at distant stage was higher among black women than among women of other racial/ethnic groups, overall (16% versus 8%–10%) and for each histologic type, particularly sarcoma (45% versus 30%–34%).

**TABLE:** Number and rate* of invasive uterine cancer cases (2015) and deaths (2016),^†^ by selected characteristics — United States^§^

Characteristic	Overall		Racial/Ethnic group^¶^									
			White		Black		American Indian/ Alaska Native		Asian/Pacific Islander		Hispanic	
	No. (%)	Rate	No. (%)	Rate	No. (%)	Rate	No. (%)	Rate	No. (%)	Rate	No. (%)	Rate
**Incidence**	53,911 (100)	26.5	39,768 (100)	27.0	6,105 (100)	26.5	324 (100)	23.1	2,053 (100)	19.2	5,114 (100)	23.2
Stage at diagnosis**												
Localized	36,021 (67)	17.7	27,393 (69)	18.7	3,359 (55)	14.5	219 (68)	15.6	1,369 (67)	12.8	3,395 (66)	15.2
Regional	11,273 (21)	5.5	8,144 (20)	5.4	1,506 (25)	6.5	64 (20)	4.6	424 (21)	4.0	1,057 (21)	4.9
Distant	4,698 (9)	2.3	3,010 (8)	2.0	997 (16)	4.4	26 (8)	1.9	196 (10)	1.9	449 (9)	2.2
Unknown	1,919 (4)	1.0	1,221 (3)	0.8	243 (4)	1.1	15 (5)	1.1	64 (3)	0.6	213 (4)	1.0
**Histologic type**												
Endometrioid carcinoma	36,425 (68)	17.9	28,261 (71)	19.3	2,870 (47)	12.4	219 (68)	15.8	1,386 (68)	12.9	3,351 (66)	14.9
Other carcinoma	12,676 (24)	6.1	8,685 (22)	5.7	2,032 (33)	8.8	79 (24)	5.4	477 (23)	4.5	1,224 (24)	5.8
Carcinosarcoma	2,714 (5)	1.3	1,625 (4)	1.0	719 (12)	3.1	13 (4)	1.0	85 (4)	0.8	259 (5)	1.3
Sarcoma	1,790 (3)	1.0	1,013 (3)	0.8	425 (7)	1.9	9 (3)	0.6	92 (4)	0.9	237 (5)	1.0
**Histologic type diagnosed at distant stage**												
Endometrioid carcinoma	1,124 (3)	0.6	845 (3)	0.6	135 (5)	0.6	7 (3)	0.5	53 (4)	0.5	81 (2)	0.4
Other carcinoma	2,288 (18)	1.1	1,452 (17)	0.9	500 (25)	2.2	14 (18)	1.0	88 (18)	0.9	219 (18)	1.1
Carcinosarcoma	609 (22)	0.3	353 (22)	0.2	163 (23)	0.7	—^††^	—	25 (29)	0.2	65 (25)	0.3
Sarcoma	643 (36)	0.3	339 (33)	0.3	191 (45)	0.9	—	—	28 (30)	0.3	81 (34)	0.4
**Deaths**	10,733	5.0	7,391	4.6	2,048	9.0	52	3.7	378	3.5	841	4.0

**Sources:** CDC’s National Program of Cancer Registries; National Cancer Institute’s Surveillance, Epidemiology, and End Results program; and CDC's National Center for Health Statistics National Vital Statistics System.

* Per 100,000 women, age-adjusted to the 2000 U.S. standard population.

^†^ Uterine cancer cases were defined as microscopically confirmed cancers of the corpus uteri (*International Classification of Diseases for Oncology, Third Edition* [ICD-O-3] site codes C54.0 [isthmus uteri], C54.1 [endometrium], C54.2 [myometrium], C54.3 [fundus uteri], C54.8 [overlapping lesion of corpus uteri], C54.9 [corpus uteri]), and uterus, not otherwise specified (C55.9), excluding cases that were identified by autopsy or death certificate only. Only cases defined as malignant under *International Classification of Diseases for Oncology, Second Edition* (ICD-O-2) and ICD-O-3 were included in this report. Histologic types were classified by ICD-O-3 histology codes, and include endometrioid carcinomas (8380); other carcinomas (8000–8379, 8381–8790, 8981, 9700–9701); carcinosarcomas (8950 [Müllerian tumors], 8951, 8980); and sarcomas (8800–8932, 8934–8941, 8959–8975, 9141–9582). Uterine cancer deaths were defined as deaths from cancers of corpus uteri (*International Classification of Diseases 10th Edition* [ICD-10] codes C54.0–C54.3, C54.8, C54.9) and uterus, not otherwise specified (C55.9).

^§^ Cancer incidence compiled from cancer registries that meet the data quality criteria in 2015, covering 100% of the U.S. population. Cancer mortality data cover 100% of the U.S. population.

^¶^ Mutually exclusive racial/ethnic groups are based on information about race/ethnicity that was collected separately and combined for this report. White, black, American Indian/Alaska Native, and Asian/Pacific Islander race categories are all non-Hispanic. Hispanic persons can be any race. Data are not presented for those with unknown or other race or unknown ethnicity.

** A localized cancer is one that is confined to the primary site, a regional cancer is one that has spread directly beyond the primary site or to regional lymph nodes, and a distant cancer is one that has spread to other organs.

^††^ Dashes indicate that statistic could not be calculated because fewer than six cases were reported.

During 1999–2015, uterine cancer incidence rates increased 12%, about 0.7% per year on average, with larger increases observed among AI/AN (53%; AAPC = 2.7%), black (46%; 2.4%), API (38%; 2.0%), and Hispanic (32%; 1.8%) women than among white women (9%; 0.5%) (Figure 1). During 1999–2015, incidence rates of endometrioid carcinomas increased 4.5% per year, other carcinomas decreased 4.5% per year, carcinosarcomas increased 1.9% per year, and sarcoma incidence remained stable (Supplementary Figure, https://stacks.cdc.gov/view/cdc/60809).

**FIGURE 1:**
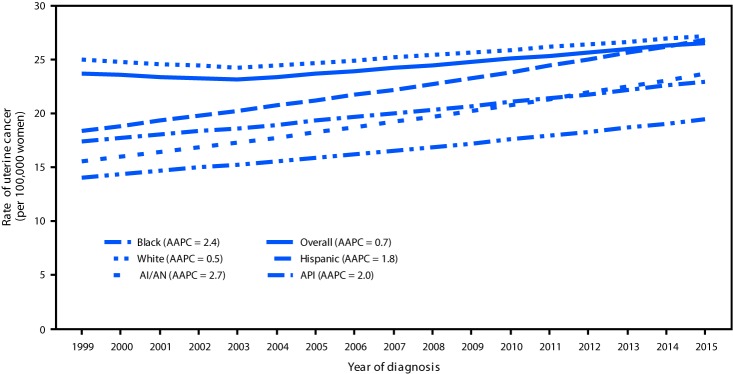
Trends* in age-adjusted uterine cancer incidence rates,**^†^** by racial/ethnic group**^§^** — United States,**^¶^** 1999–2015 **Sources:** CDC’s National Program of Cancer Registries and the National Cancer Institute’s Surveillance, Epidemiology, and End Results program. **Abbreviations:** AAPC = average annual percent change; AI/AN = American Indian/Alaska Native; API = Asian/Pacific Islander. * Trends were measured with AAPC in rates and were considered to increase or decrease if p<0.05; otherwise, rates were considered stable. AAPC is the weighted average of the annual percent change over the period 1999–2015 using a Joinpoint regression model (up to 2 joinpoints). ^†^ Per 100,000 women, age-adjusted to the 2000 U.S. standard population. Uterine cancers were defined as microscopically confirmed cancers of the corpus uteri (*International Classification of Diseases for Oncology, Third Edition* [ICD-O-3] site codes C54.0–C54.3, C54.8, C54.9) and uterus, not otherwise specified (C55.9), excluding cases that were identified by autopsy or death certificate only. ^§^ Mutually exclusive racial/ethnic groups are based on information about race/ethnicity that was collected separately and combined for this report. White, black, AI/AN, and API race categories are all non-Hispanic. Hispanic persons can be any race. ^¶^ Cancer incidence compiled from cancer registries that meet the data quality criteria for each year during the period 1999–2015, covering 98% of the U.S. population.

In 2016, 10,733 uterine cancer deaths, corresponding to five deaths per 100,000 women, were reported in the United States (Table). Uterine cancer death rates among black women (nine deaths per 100,000) were higher than those among white (five), AI/AN (four), API (four), and Hispanic (four) women. During 1999–2016, uterine cancer death rates increased 21%, approximately 1.1% per year on average, with larger increases among API (52%; AAPC = 2.5%), Hispanic (33%; 1.7%), and black (29%; 1.5%) women, than among white women (18%; 1.0%); rates were stable among AI/AN women (Figure 2).

**FIGURE 2:**
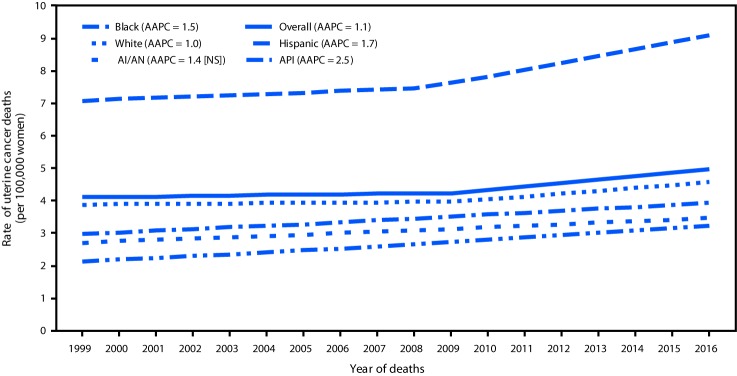
Trends* in age-adjusted uterine cancer death rates,**^†^** by racial/ethnic group**^§^** — United States, 1999–2016 **Source:** CDC’s National Center for Health Statistics National Vital Statistics System. **Abbreviations:** AAPC = average annual percent change; AI/AN = American Indian/Alaska Native; API = Asian/Pacific Islander; NS = not significant. * Trends were measured with AAPC in rates and were considered to increase or decrease if p<0.05; otherwise rates were considered stable. AAPC is the weighted average of the annual percent change over the period 1999–2016 using a Joinpoint regression model (up to 2 joinpoints). ^†^ Per 100,000 women, age-adjusted to the 2000 U.S. standard population. Uterine cancer deaths were defined as deaths from cancers of corpus uteri (*International Classification of Diseases 10th Edition* [ICD-10] codes C54.0–C54.3, C54.8, C54.9) and uterus, not otherwise specified (C55.9). ^§^ Mutually exclusive racial/ethnic groups are based on information about race/ethnicity that was collected separately and combined for this report. White, black, AI/AN, and API race categories are all non-Hispanic. Hispanic persons can be any race.

## Discussion

This report indicates that the rate of new uterine cancer cases increased during 1999–2015, with larger increases observed among black, AI/AN, API, and Hispanic women than among white women. This contrasts with the recent decreases in incidence rates that have been observed for many cancer types, such as lung and colorectal cancers (*1*). One contributing factor to increasing uterine cancer incidence could be excess body weight; women who are overweight (body mass index [BMI] = 25.0–29.9 kg/m^2^) or have obesity (BMI ≥30 kg/m^2^) are approximately two to four times as likely to develop endometrial cancer as are women with healthy weight (*4*). During 2013–2016, approximately 40% of women in the United States had obesity, including 56% of black women and 49% of Hispanic women.*** The U.S. Preventive Services Task Force recommends that clinicians offer or refer adults with obesity to intensive, multicomponent behavioral interventions.^†††^ Community-based strategies to promote healthy body weight include helping persons meet dietary and physical activity guidelines by supporting healthy eating and active living in such settings as communities, worksites, schools, and early care and education facilities (*4*). Other factors such as insufficient physical activity, increasing prevalence of diabetes, and decreasing use of estrogen plus progestin menopausal hormone therapy might also contribute to increases in endometrial cancer incidence (*5*).

This report also found that uterine cancer death rates were higher in 2016 than in 1999 and that black women were approximately twice as likely to die from uterine cancer as were women in other racial/ethnic groups. As with other cancers, the odds of surviving uterine cancer are much higher when it is detected at an early stage, when treatment is more effective (*5*). The 5-year relative survival estimate for localized uterine cancer is 80%–90% compared with <30% for distant uterine cancer (*5*). This report found that black women were more likely to receive a diagnosis at distant stage and with more aggressive histologic types than were other women, which might in part account for the higher death rate among black women.

Although population-based screening tests are recommended for several cancers, including breast, cervical, colorectal, and lung cancers, at present, population-based screening tests are not recommended for uterine cancer (*6*). An important early symptom of uterine cancer is abnormal vaginal bleeding, including bleeding between periods or after sex or any unexpected bleeding after menopause (i.e., any bleeding except intermittent bleeding within 1 year after cessation of menses or cyclic bleeding associated with use of cyclic postmenopausal hormone therapy) (*3*). Approximately 90% of women with uterine cancer report abnormal vaginal bleeding (*6*). A lower percentage of women with uterine sarcomas have abnormal vaginal bleeding (approximately 56%) or nonspecific symptoms, such as pelvic pain (22%); consequently, a higher percentage of sarcomas are not detected until the cancer has already spread (*7*). Uterine cancer outcomes could be improved by increasing awareness among women that abnormal vaginal bleeding should be evaluated promptly by a health care provider. It is also important for health care providers to perform timely evaluation and necessary follow-up of women’s concerns and symptoms (*8*). Transvaginal ultrasonography or endometrial tissue sampling are appropriate for initial evaluation of postmenopausal bleeding; further evaluation could include hysteroscopy combined with endometrial sampling (*8*). To help women make informed choices, health care providers can educate women about different procedural options (including surgical choices); discuss the benefits and risks of each procedure; and discuss the risk for cancer (*9*). CDC’s Inside Knowledge campaign attempts to raise awareness among women and health care providers about uterine cancer and other gynecologic cancers. Inside Knowledge uses a multimedia approach to ensure campaign messages reach the broadest audience possible.

The findings in this report are subject to at least five limitations. First, reporting of race and ethnicity uses data from medical records and death certificates, which might be inaccurate in some cases, especially among AI/AN; ongoing procedures are used to ensure that this information is as accurate as possible.^§§§^ Second, improved pathologic classification of tumors over time might influence rates and trends. Third, broad groups were used for histologic type, which might mask varying levels of tumor behavior. Fourth, in clinical practice, uterine cancers are commonly staged on the basis of histologic type using the International Federation of Gynecology and Obstetrics system (*6*); however, because this information is not routinely collected in cancer registries, this report used SEER Summary Stage to stage cancers. Finally, rate denominators were not adjusted for hysterectomy prevalence and might include women who did not have an intact uterus and were not at risk for uterine cancer, thus underestimating the actual rate among women at risk, particularly black women, who have higher rates of hysterectomy (*10*).

Multifactorial efforts at individual, community, clinical, and systems levels to help women achieve and maintain a healthy weight and obtain sufficient physical activity might reduce the risk for developing uterine cancer. Promoting awareness among women and health care providers of the need for timely evaluation of abnormal vaginal bleeding can increase the chance that uterine cancer is detected early and treated appropriately.

**Summary**

**What is already known about this topic?**

Uterine cancer is one of the few cancers with increasing incidence and mortality.

**What is added by this report?**

During 1999–2015 and 1999–2016, uterine cancer incidence and mortality rates increased 0.7% and 1.1% per year, respectively, with black women disproportionately affected.

**What are the implications for public health practice?**

Health care providers and community programs can help women achieve and maintain a healthy weight and get enough physical activity, which can reduce the risk for endometrial cancer, the most common uterine cancer. Promoting awareness of the need for timely evaluation of abnormal vaginal bleeding (between periods, after sex, or after menopause), an important symptom of uterine cancer, increases the chance for early detection and treatment.

## Footnotes

* https://www.cdc.gov/cancer/knowledge/.

† Uterine cancer cases were defined as microscopically confirmed invasive cancers of the corpus uteri (*International Classification of Diseases for Oncology, Third Edition* [ICD-O-3] site codes C54.0 [isthmus uteri], C54.1 [endometrium], C54.2 [myometrium], C54.3 [fundus uteri], C54.8 [overlapping lesion of corpus uteri], C54.9 [corpus uteri]) and uterus, not otherwise specified (C55.9), excluding cases that were identified by autopsy or death certificate only. Only cases defined as malignant under *International Classification of Diseases for Oncology, Second Edition* (ICD-O-2) and ICD-O-3 were included in this report. Uterine cancer deaths were defined as deaths from cancers of corpus uteri (*International Classification of Diseases for Oncology, Tenth Edition* [ICD-10] codes C54.0–C54.3, C54.8, C54.9) and uterus, not otherwise specified (C55.9).

§ https://www.cdc.gov/cancer/uscs/index.htm.

¶ Cancer registries’ incidence data met the following U.S. Cancer Statistics criteria: 1) ≤5% of cases ascertained solely on the basis of death certificate; 2) ≤3% of cases missing information on sex; 3) ≤3% of cases missing information on age; 4) ≤5% of cases missing information on race; and 5) ≥97% of registry’s records passed a set of single-field and interfield computerized edits that test the validity and logic of data components. https://www.cdc.gov/cancer/npcr/uscs/technical_notes/criteria.htm.

§§§ https://www.cdc.gov/cancer/npcr/uscs/technical_notes/interpreting/race.htm.
